# LncRNA SNHG6 Silencing Could Arrest Progression of High Grade Colorectal Cancers

**DOI:** 10.30699/IJP.2021.527781.2610

**Published:** 2021-12-15

**Authors:** Amin Jafari-Oliayi, Shahriar Dabiri, Malek Hossein Asadi

**Affiliations:** 1 Pathology and Stem Cell Research Center, Department of Pathology, Afzalipour Medical School, Kerman University of Medical Sciences, Kerman, Iran; 2 Institute of Science and High Technology and Environmental Sciences, Graduate University of Advanced Technology Kerman, Iran

**Keywords:** Cell cycle, Colorectal cancer, Long non-coding RNAs

## Abstract

**Background & Objective:**

Colorectal cancer (CRC), like other cancers, needs faster and more accurate identifications. A well-timed prognosis of CRC could be an important turning point in the survival of patients. Supplementary signs, such as long non-coding RNAs (lncRNAs), could be helpful for this purpose. A new possible biomarker for CRC identification is introduced by this study.

**Methods:**

RNA extraction was performed by the RNX-Plus solution for 64 tumor and non-tumor tissues. Complementary DNAs (cDNAs) were synthesized, and quantitative real-time PCR was performed for relative expression level measurement and the data was analyzed statistically using the Prism 6 software. For Small nucleolar host gene 6 knockdown, siRNA was designed based on Reynolds rules. The cells were cultured in their appropriate media, and the siRNA-lipofectamine complex was formed. The transfection complex was presented for sw48, sw480, and sw1116 as CRC cells with different grades. After transfection, the SNHG6/β actin ratio was determined. Then, the distribution of siRNA-treated cells was determined by the Partec flow cytometer instrument and analyzed by the FloMax software.

**Results:**

SNHG6 was more expressed in CRC tumors than non-tumor tissues. In tumor tissues, SNHG6 upregulation and tumors’ grade progression were concurrent. SNHG6 was upregulated in cases with lymphovascular invasion than in cases with perineural invasion. The knockdown of SNHG6 conduced to G1 arrest in CRC cells, more noticeably in high-grade ones.

**Conclusion:**

SNHG6 could be applied as a consideration to differentiate tumor and non-tumor tissues and grade definition in colorectal malignancies, and it could participate in colorectal tumor formation as a cell cycle progressive factor.

## Introduction

Cancer is a complicated disease accompanied by genetic and/or metabolic causes (1, 2). Colorectal cancer (CRC) is one of the frequent cancers (3). Like many cancers, CRC has a specific expression pattern of long non-coding RNAs (lncRNAs) in its transcriptome. Non-coding RNAs (ncRNAs) as new players of cancer progression have become increasingly interested in the last decade; lncRNAs are important and famous members of this category (4). 

It has been proved that many tumor tissues alter the expression level of lncRNAs compared to their respective non-tumor marginal tissues. Thus, the expression of these transcripts could be an important sign for prognosis and diagnosis of cancers (5-7).

Some lncRNAs are upregulated in CRC. These RNAs act via different pathways for the progression of cancer in colorectal tissue (8). One of the lncRNA families that affect the colorectal tissue is the small nucleolar host gene (SNHG) family. This family has many members, such as SNHG1, SNHG3, SNHG5, SNHG7, SNHG12, SNHG15, and SNHG20. The important characteristic of this family is the presence of short sequences of small nucleolar RNAs (snoRNAs) in their sequence. Because of this characteristic, they are called snoRNA host genes (9). 

**Table 1:** Number, percentage and some clinicopathological characteristics of the tumor samples with detectable SNHG6 expression level are shown

Characteristic	Number
Grade I	**10 (31.2 %)**
Grade II	**9 (28.1 %)**
Grade III	**5 (15.6 %)**
Grade IV	**3 (9 %)**
Tumors with unknown grade	**5 (15.6 %)**
Stage II	**12 (37.5 %)**
Stage III	**12 (37.5 %)**
Stage IV	**2 (6 %)**
Tumors with unknown stage	**6 (18.7 %)**
Tumor size	
≤ 6 cm	**11 (34.3 %)**
>6 cm	**12 (37.5 %)**
Tumors with unknown size	**9 (28 %)**
Gender	
Male	**16 (50 %)**
Female	**10 (31.2 %)**
Patients with unknown sex	**6 (18.7 %)**
Age	
≤ 60	**11 (34.3 %)**
>60	**12 (37.5 %)**
Patients with unknown age	**9 (28 %)**
Invasions	
Pre-neural	**4 (12.5 %)**
Lympho-vascular	**12 (37.5 %)**
Tumors with unknown invasion site	**16 (50 %)**

Further, snoRNAs have 65-300 nucleotides in length and are usually transcribed as a polycistronic unit. Usually, they have no poly-A tail and 5' cap; snoRNAs are divided into two categories with different functions. CD box and H/ACA box snoRNAs are two kinds of snoRNAs (10-12).

LncRNAs are located in the nucleus and/or cytoplasm (13). The cytoplasm or nucleus location of these RNAs is one of the most important items, determining the function of these transcripts. The cytoplasmic location of these RNAs could relate to microRNA (miRNA) sponging, binding to messenger RNAs (mRNAs), and repression of their translation and protein ubiquitination inhibition. Their nucleus location could relate to DNA methylation and transcription inhibition. SNHGs could act differentially based on their location (12). 

SNHG family members function via different molecular pathways to alter cellular behaviors. For instance, SNHG1 could interact with the mediator complex of solute carrier family 3 member 2 (SLC3A2) as a cancer-promoting factor and progress gastric and osteosarcoma malignancies (14-16). 

SNHG6 is one of the SNHG family members and has been discussed in many studies as oncogenic lncRNA (17-20). SNHG6 contains small nucleolar RNA C/D box 87 (U87 snord) in one of its introns, known as the U87 host gene (9). 

In this research, we focused on the SNHG6 expression pattern of CRC tumors and non-tumor tissues. Also, the SNHG6 expression level was studied in different grades of tumor samples. Finally, following SNHG6 knockdown, the cell cycle progression of CRC cells with different grades was studied.

## Material and Methods

**Sample Collection**

Sixty-four tissues (32 tumor and 32 adjacent non-tumor tissues) from 32 CRC patients, as well as their respective datasheets, were received from Imam Khomeini Cancer Institute (Tehran, Iran). The tissues were stored in liquid nitrogen until they were used for RNA extraction. Number, percentage, and some clinicopathological characteristics of the tumor samples that had detectable SNHG6 expression levels are shown in Table 1. As a limitation of our research, some datasheets had incomplete information.

**RNA Extraction and Complementary DNA Synthesis**

RNA extraction was performed by the RNX-Plus solution (SinaGen, Iran). Available tissues were frozen in liquid nitrogen and then ground in the presence of the RNX-Plus solution as a preservative. Also, 200 µL of chloroform (Merck, Germany) was added to each micro-tube and shacked vigorously for 15 seconds. Micro-tubes were placed on ice for 15 minutes then centrifuged for 15 minutes at 12 000 rpm and 4ºC. The upper layers of tubes were transferred to new micro-tubes, and 500 µL of isopropyl alcohol (Merck, Germany) was added to each tube and mixed gently. The micro-tubes were placed at -20ºC for 45 minutes then centrifuged as explained above. The RNA pellet was dissolved in 30 µL of ribonuclease (RNase) free water and stored at -70ºC. Respective complementary DNAs (cDNAs) were synthesized as the protocol of cDNA synthesis of the Fermentas cDNA Kit (USA).

**Quantitative real-time polymerase chain reaction**

The SNHG6 expression level was measured by the ABI instrument (Life Technology, USA), and the data were normalized using β Actin as an appropriate housekeeping gene. Relative expression was calculated based on the ΔΔCt method. We designed primers based on primer design rules using the GeneRunner software. We used TB Green Premix Ex Taq II (Takara Bio, Japan) for assessing the relative expression of target variants. The ABI real-time instrument (Life Technology) and its software were used for the measurement of relative gene expression. Each assay was replicated two times. The primers sequences and Q-real time PCR condition were mentioned in Table 2 and Table 3**.**

**Table 2:** Sequences of the primers that were used in the study

Primer	? 5’ 3’ sequence
β Actin F	**ACTCTCTTCCAGCCTTCCTTCCT**
β Actin R	**ACTGACAGCACTGTGTTGGCGTA**
SNHG6 F	**GACCGGCGAGGGAGGAAGAA**
SNHG6 R	**CGCAGAGCCCAGCTACGG**

**Table 3:** The condition of Q-real time PCR for expression level measurement of lncRNA SNHG6

	Initial denaturation	Cycle denaturation	Annealing and extension	Number of Cycle	Annealing temperature
β Actin	40 s	5 s	30 s	40	60ºc
SNHG6	40 s	5 s	30 s	40	63ºc

Small Interfering RNA Design 

Small interfering RNA (siRNA) for SNHG6 knockdown was designed based on Reynolds rules (21) and purchased from the Gene-Fanavaran Company (Iran).

Cell Culture and Transfection

The cells (sw48, sw480, and sw1116) were cultured in RPMI and DMEM media (Gibco, UK) at 37ºC and 5% CO_2_ in the humidified Memmert Incubator. The cells were transfected by lipofectamine 2000 as done before (17). Based on the Broders classification, sw48 and sw480 are grade 4, and sw1116 is grade 2 (22). 

Cell Cycle Analysis

The preparation of cells for flow cytometry was done according to Alipoor and Keshavarz *et al.* (23, 24). The percentage of cells in three phases of the cell cycle was identified using the Partec flow cytometer instrument (Germany), and the gained data were analyzed by the FloMax software (Germany). Briefly, the cultured cells were harvested using trypsin, washed in phosphate-buffered saline (PBS), and stained with a 50-μg/mL propidium iodide (PI) solution containing 0.1% Triton X-100 and 0.1% sodium citrate. Then, the cells were counted by the flow cytometer instrument and analyzed by FloMax software. 

Statistical Analyses

We used the unpaired *t*-test and analysis of variance (ANOVA) for statistical analyses. All gained data were statistically analyzed using the Prism 6 software package, and P-value < 0.05 was considered statistically significant.

## Results

**More Expression of SNHG6 in Tumor Tissues than Non-tumors**

**Fig. 1:**
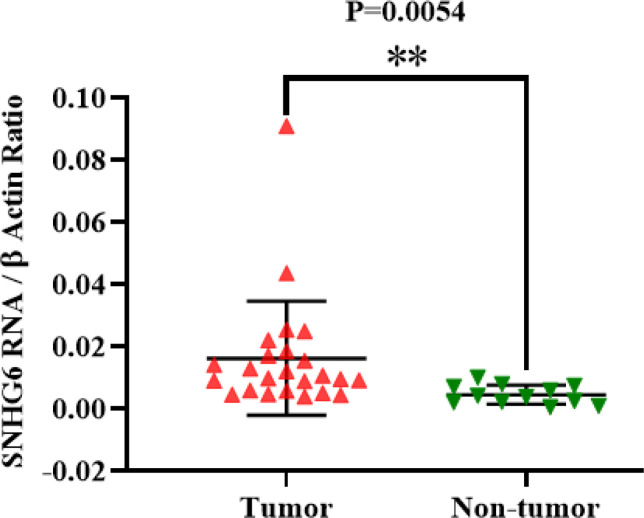
SNHG6 was expressed in colorectal tumor tissues higher than non-tumor ones significantly

First, it should be mentioned that we had 32 tumor tissues, and some of them had incomplete clinicopathological data. The expression of SNHG6 RNA was measured in 32 tumor tissues. Some of these 32 tumor tissues had no detectable SNHG6 expression; thus, they were excluded from statistical analyses. Q-real time PCR data demonstrated that the lncRNA SNHG6 expression level was higher in colorectal tumor tissues than non-tumor marginal tissues. This difference was significant (*P*=0.0054; Figure 1). 

More Expression of SNHG6 in High-Grade Colorectal Tumors than Low-Grade ones

**Fig. 2:**
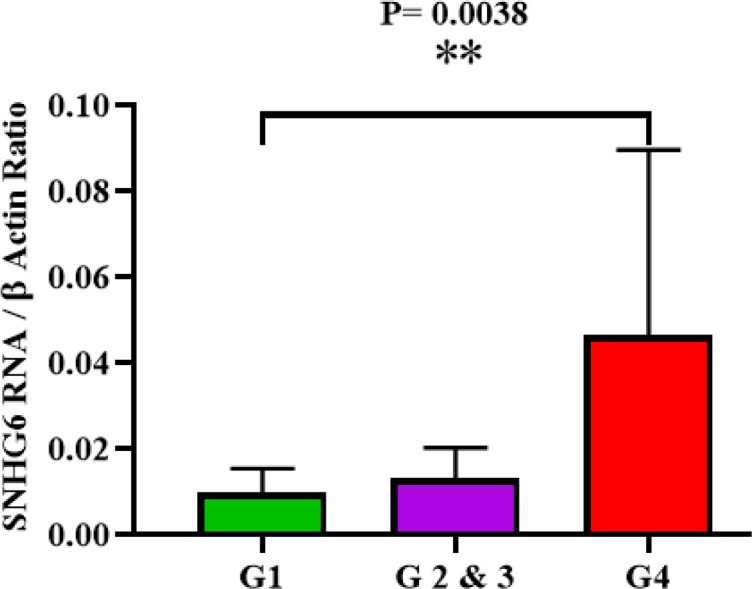
SNHG6 was expressed in high grade colorectal cancer tumors more than low grade ones

Analyzed expression data demonstrated that tumor grade progression of studied patients and higher expression levels of SNHG6 RNA in their tumors were concurrent. The difference of SNHG6 expression level in grades was significant P-value= 0.0038 (Figure 2).

**Higher SNHG6 Content in Tumors with Lymphovascular Invasion than Tumors with Perineural Invasion**

**Fig. 3:**
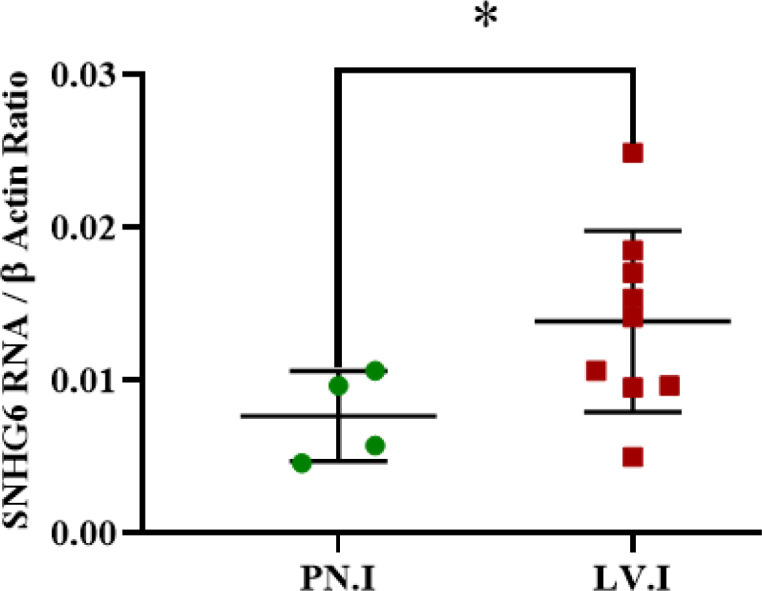
SNHG6 was expressed in tumors with lympho-vascular invasions more than tumor tissues with perineural invasion

Analyzed expression data showed that patients with lympho-vascular invasion had more SNHG6 expression levels than those with perineural invasion in their tumors (Figure 3; Table 4).

**Table 4:** SNHG6 expressions measurement in some clinicopathological characteristics of the tumor samples and its statistical comparison

Characteristics	Number (%)	Expression mean ± SD
Grade Grade I Grade II & III Grade IV	8 (25%) 13 (40.6%) 3 (9%)	**0.0097 ± 0.0055** **0.013 ± 0.007** **0.0463 ± 0.0432**
Tumor Non-tumor	24 (75 %) 12 (37.5%)	**0.0114 ** **±** ** 0.0066** **0.0034 ± 0.0020**
Perineural invasion Lympho-vascular involvement	**4 (12.5 %)** **9(28.1 %)**	**0.0076 ± 0.0029** **0.0138 ± 0.0059**

**Significant higher SNHG6 Expression in ≤6 cm Tumors than >6 cm Tumors**

**Fig. 4:**
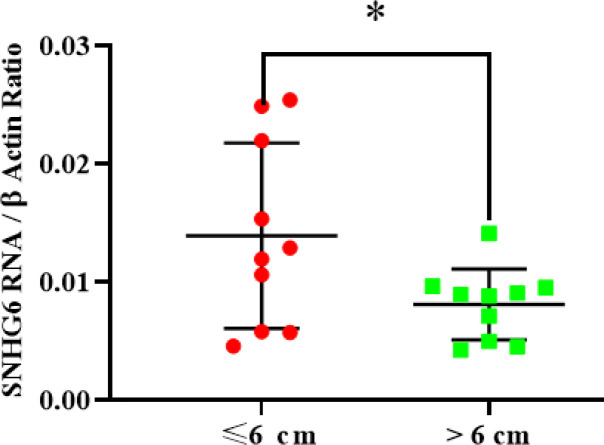
SNHG6 was expressed in **≤**6 cm tumors more than >6 cm tumors significantly

SNHG6 expression level measurement demonstrated that ≤6-cm tumors had more SNHG6 content compared to >6-cm tumors. The difference was significant (Figure 4).

**Patients Aged over 60 with a Significant High Expression of SNHG6**

**Fig. 5:**
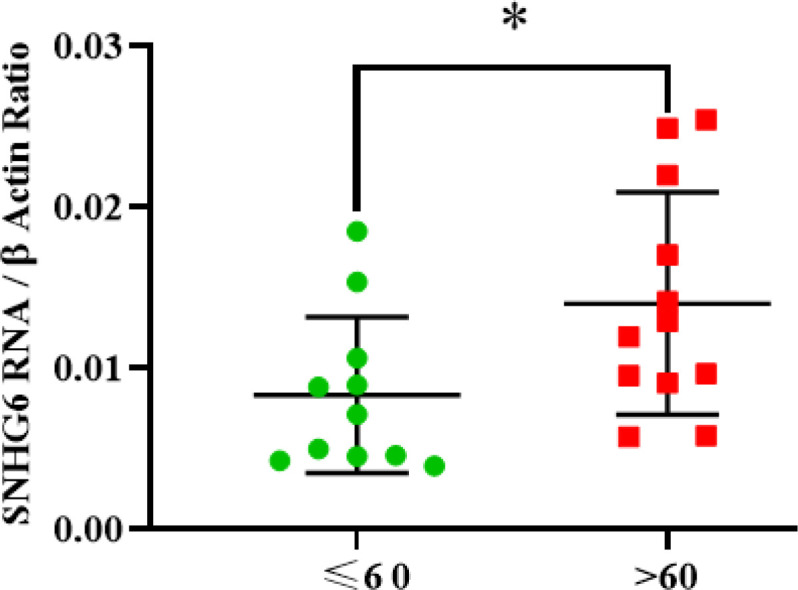
SNHG6 was expressed in patients aged over 60 more than patients were 60 and under 60 years old. The difference was significant

SNHG6 expression level measurement demonstrated that patients over 60 showed higher expression levels of SNHG6 in their tumor tissues compared to the patients aged 60 or younger (Figure 5). 

**Irrelevance of SNHG6 Expression Level in Tumor Tissues and Patient’s Gender**

**Fig. 6:**
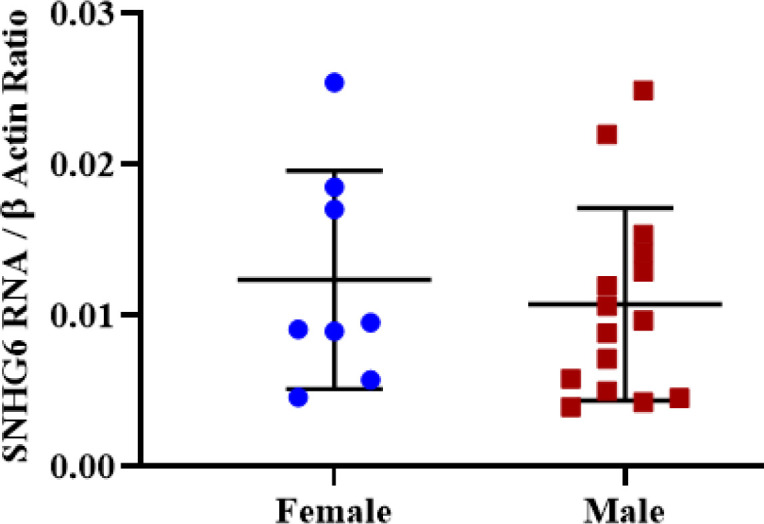
Expression was not different significantly in tumor tissues of male and female patients

No significant expression difference was observed between male and female tumors after SNHG6 expression measurement (Figure 6).

**Non-significant Difference of SNHG6 Expression Level in Different Stages of CRC**

**Fig. 7:**
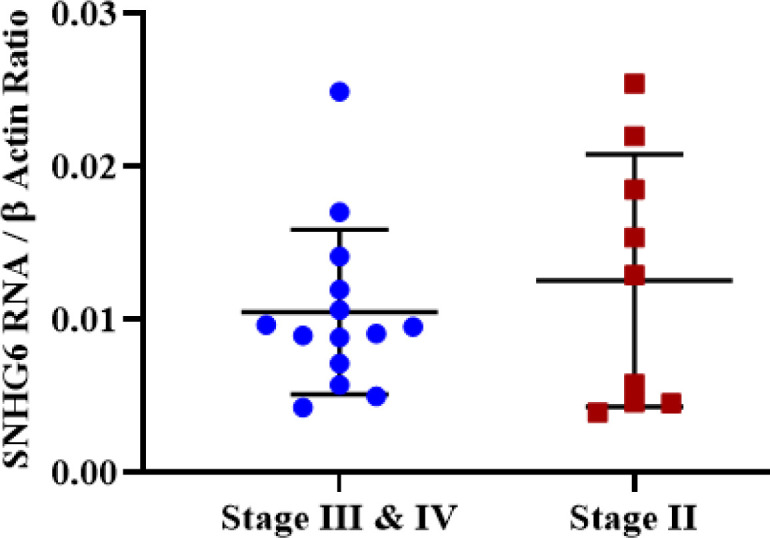
Tumor samples with different stages did not demonstrate a significant expression difference of SNHG6

 Regarding the SNHG6 expression level, no significant difference was observed between different stages of tumor samples (Figure 7). 

**Coincidence of SNHG6 Knockdown and Significant G1 Arrest of High-Grade CRC Cells**

**Fig. 8:**
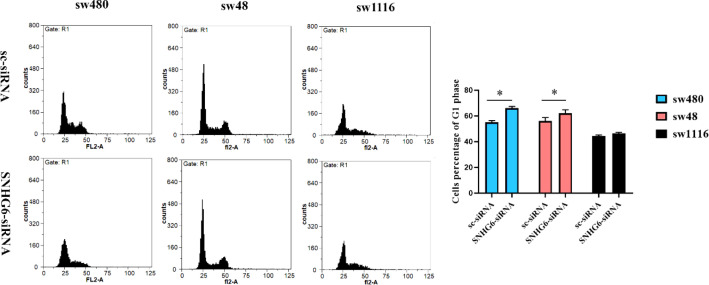
Following SNHG6 knockdown, sw480 and sw48 cells as high-grade colorectal cancer cells demonstrated a significant G1 arrest. Sw1116 cells as low-grade colorectal cancer cells demonstrated no significant G1 arrest following SNHG6 silencing

All three studied CRC cell lines (sw480, sw48, and sw1116) demonstrated G1 arrest after SNHG6 RNA knockdown. This event was not equal in all three cell lines but was happened in all of the target cells. High-grade CRC cells (sw480 and sw48) significantly exposed obvious G1 arrest following SNHG6 silencing. On the other hand, sw1116 cells (as low-grade cells) had no significant and pronounced G1 arrest in the same situation (Figure 8).

## Discussion

Many lncRNAs have been propounded as upregulated transcripts in different tumor tissues. SNHG6 RNA (like many lncRNAs) has an upregulated expression level in many cancers (17, 20, 24). 

Consistent with previous studies, we observed that lncRNA SNHG6 was upregulated in colorectal tumor tissues compared to non-tumor marginal tissues (Figure 1) (25-27). Previous studies have shown that the expression level of SNHG6 RNA is higher in colorectal tumor tissues than in non-tumor tissues, indicating lncRNA SNHG6 as a valuable prognostic biomarker. 

Grade progression of tumors (like many aspects of tumor tissues) could be affected by lncRNAs (28). In this research, high-grade CRC tissues demonstrated a higher expression level of SNHG6 RNA than low-grade tissues (Figure 2) (Table 4). A higher expression level of SNHG6 in more undifferentiated CRC cells might be a sign of involvement of SNHG6 in CRC cell stemness state. Actually, coincident reduction of SNHG6, Octamer-binding transcription factor 4 (OCT4), and Nanog was proved in differentiating NCCIT cells (17). Altogether, lncRNA SNHG6 could be contributed to the differentiation and grade progression of CRC cells. 

Determination of the SNHG6 expression pattern in patients with different invasion sites indicated that the SNHG6 expression level was higher in patients with lymphovascular invasion than patients with perineural invasion, and the difference was significant (Figure 3). This observation could be construed that SNHG6 might be a powerful tool for CRC cells to invade distant tissues (29). 

Nowadays, lncRNAs are known as an important factor of cell cycle progression (30). Cell cycle progression is one of the basic points of cells survival, especially for cancer cells. Many cancer treatments are based on drugs that stop the cell cycle progression of cancer cells (31-33). Each factor that disrupts the cell cycle progression of cancer cells could be a hope for the design of new drugs. Some lncRNAs participate in the progression of the cancer cell cycle (34-36). CRC (like many other cancers) is affected by lncRNAs, and these transcripts could have a crucial role in CRC progression (37). Several studies have demonstrated the relationship of lncRNA SNHG6 and cell cycle progression of CRC cells (38, 39), but none of them have discussed the interconnection of CRC grades and SNHG6 as a cell cycle progressive factor. Therefore, we chose three CRC cell lines (sw480, sw48, and sw1116), with different grades based on the Broders classification (22). SNHG6 knockdown in high-grade CRC cells (sw48 and sw480) conduced significant and obvious G1 arrest in their cell cycle. Also, knockdown data demonstrated that low-grade CRC cells (sw1116) had non-significant G1 arrest compared to high-grade ones in the same situation (Figure 8). Obvious G1 arrest in high-grade CRC cells after SNHG6 knockdown could demonstrate the critical role of SNHG6 in the cell cycle progression of more malignant colorectal cells.

## Conclusion

In conclusion, lncRNA SNHG6 could be considered as a cell cycle progressive factor of CRC cell. Its role would be more prominent in high-grade CRC cell cycle progression. In addition, SNHG6 could be considered as a novel and interesting agent for pharmaceutical industry.

## Conflict of Interest

The authors declared no conflict of interest.

## Funding

Iran National Science Foundation (INSF) has supported this research by grant number 93030186.
